# Enhancement noise margin and delay time performance of novel punch-through nMOS for single-carrier CMOS

**DOI:** 10.1186/s11671-024-04064-y

**Published:** 2024-07-06

**Authors:** Jyi-Tsong Lin, Pei-Zhang Xie, Wei-Han Lee

**Affiliations:** Department of Electrical Engineering, National Sun Yat-Sen University, Kaohsiung, 80424 Taiwan, ROC

**Keywords:** Punch-through effect, Hole mobility, Single-carrier CMOS, Noise margin, Static power dissipation, Delay time

## Abstract

In this paper, we propose the use of punch-through nMOS (PTnMOS) as an alternative to pMOS in complementary metal oxide semiconductor (CMOS) circuits. According to the TCAD simulation results, PTnMOS exhibit sub-threshold characteristics similar to those of pMOS and can be formed by simply changing the doping concentration of the source and drain. Without the need for sizing, which solves the area occupation problem caused by the need to increase the width of pMOS due to insufficient hole mobility. In addition, we compose a PTnMOS and nMOS without sizing to form a single-carrier CMOS in which only electrons are transmitted, and We extract its performance for comparison with conventional CMOS (W_p_/W_n_ = 1). The results indicate that single-carrier CMOS has symmetric noise margin and 29% faster delay time compared to conventional CMOS (W_p_/W_n_ = 1). If III–V or II–VI group materials could be applied to single-carrier CMOS, not only could costs be reduced and wafer area occupancy minimized, but also significant improvements in the performance and bandwidth application of microwave circuits could be achieved.

## Introduction

Complementary metal oxide semiconductor circuits were first proposed by Wanlass and Sah in 1963 [1] and are widely used in the modern semiconductor industry because of their extremely low static power dissipation and symmetrical noise margin. However, the mismatch of carrier mobility has always been a problem in conventional CMOS circuits. In conventional CMOS circuits, electrons and holes are the carriers of nMOS and pMOS respectively. The performance of conventional CMOS circuits is degraded since the electrons move faster than the holes, so it is necessary to make the width of the pMOS about three times wider than that of the nMOS to compensate for the problem of lower hole mobility [2], which increases both the cost of the process and the total area of layout.

Researchers have used various methods to address this. For instance, early research focused on studying different surface orientations and their effectiveness in enhancing pMOS performance [3–5]. In practice, there are limitations due to interface capture, which can compromise the reliability of the device [6]. Another method is to replace channel materials with high carrier mobility materials, such as Ge [7, 8]. However, performance degradation in ultra-thin body (UTB) Germanium on Insulator (GeOI) pMOS devices has been identified because of poor Ge/buried oxide (BOX) interfaces and GeOI thickness variations [9, 10]. Strained SOI (SSOI) has emerged as a promising option for sub-10 nm technology nodes when compared to bulk silicon and SOI technologies [11]. Strain technologies are classified as local process-induced technologies and include strained overlays [12], embedded source/drain stressors [13], stress memory technology (SMT) [14] or strained contacts and metal gates [15]. Despite the potential benefits of strain technology, its complexity can increase manufacturing costs and difficulties.

In this paper, we discuss the operating mechanism and electrical analysis of PTnMOS by TCAD simulation. Our previous studies [16, 17] have demonstrated the feasibility of the PTnMOS. However, to prevent PTnMOS from generating inversion current, it is necessary to use two embedded oxides, which significantly increases manufacturing costs and poor sub-threshold swing. Therefore, this paper proposes to use a planar fully depleted silicon-on-insulator (FD-SOI) structure to improve the control of punch-through current and inversion current switching. The planar FD-SOI structure offers better gate control, allowing it to operate at a lower supply voltage [18–20]. This enables the current to be turned off before the strong inversion occurs, so that it is not affected by the strong inversion current. Then, we present a comprehensive study and optimization of the PTnMOS is carried out. Finally, the application of single-carrier CMOS consisting of the proposed PTnMOS and nMOS in basic logic circuits is discussed.

## Structure design and fabrication

**Fig. 1:**
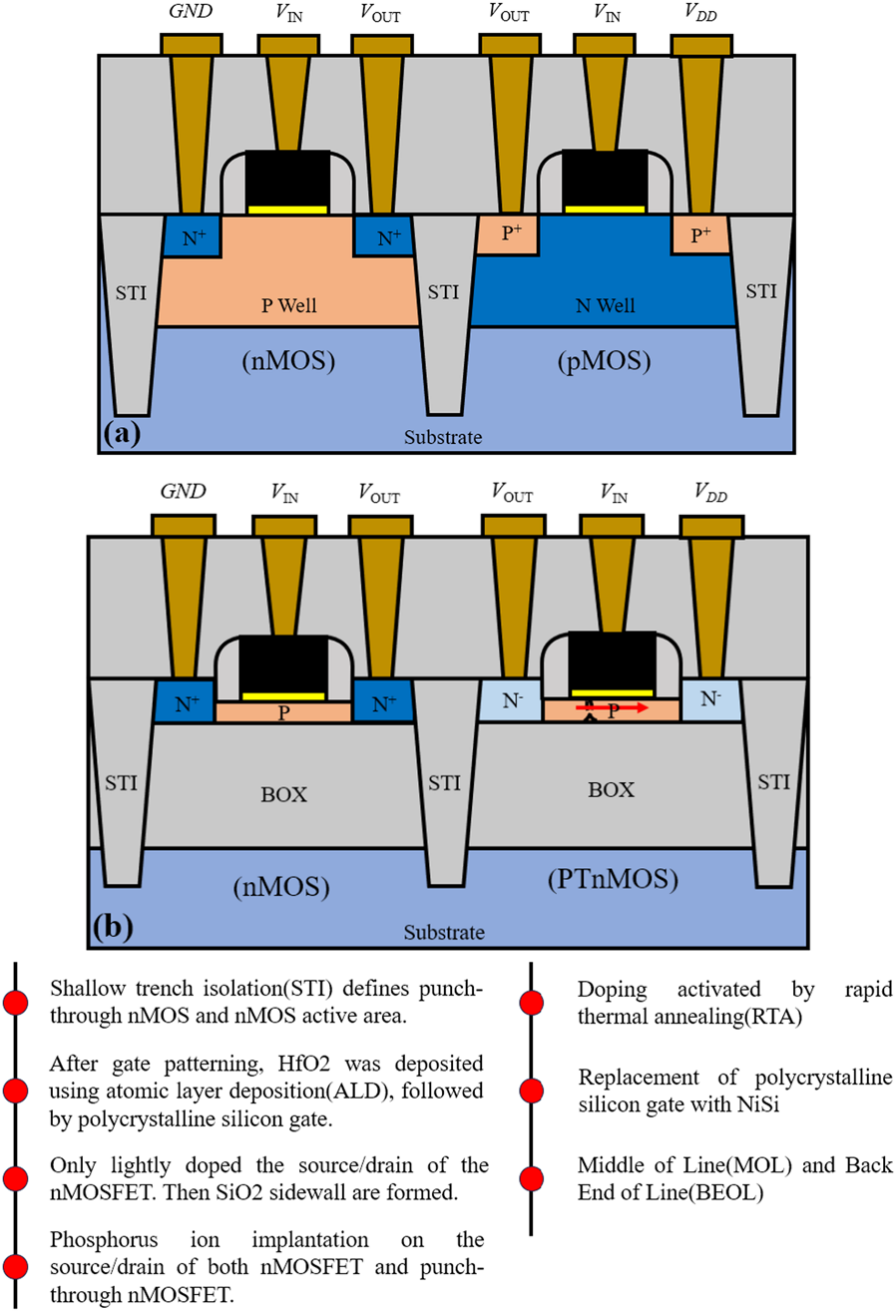
**a** Conventional CMOS structure, and **b** the proposed single-carrier CMOS structure and its main process flow

Figure 1 shows the structure of conventional CMOS circuit and our proposed single-carrier CMOS circuit, and the fabrication process of single carrier CMOS circuit. We chose channel length 22 nm because approximately 75% of the process steps are common to the 28 nm platform, improving yield capability. Firstly, in-situ doped boron 10 nm SOI and 145 nm oxygen embedded were used as initial substrates. Then shallow trench isolation (STI) is used to define the active region for PTnMOS and nMOS device. After patterning the gate structure, High-k gate dielectric (HfO_2_) is deposited by atomic layer deposition (ALD), followed by deposition of polysilicon gate. Then only lightly doped the source/drain of the nMOS. This is to avoid that lightly doped the source/drain of the PTnMOS will reduce the punch-through effect. After the formation of the SiO_2_ sidewall, phosphorus ion implantation and activation of the dopant by rapid thermal annealing (RTA) are performed on the source/drain of both nMOS and PTnMOS. Finally, the poly-Si gate is replaced with a metal gate (NiSi), followed by Middle of Line (MOL) and Back End of Line (BEOL) processes.

## Physical mechanism and simulation method

**Fig. 2:**
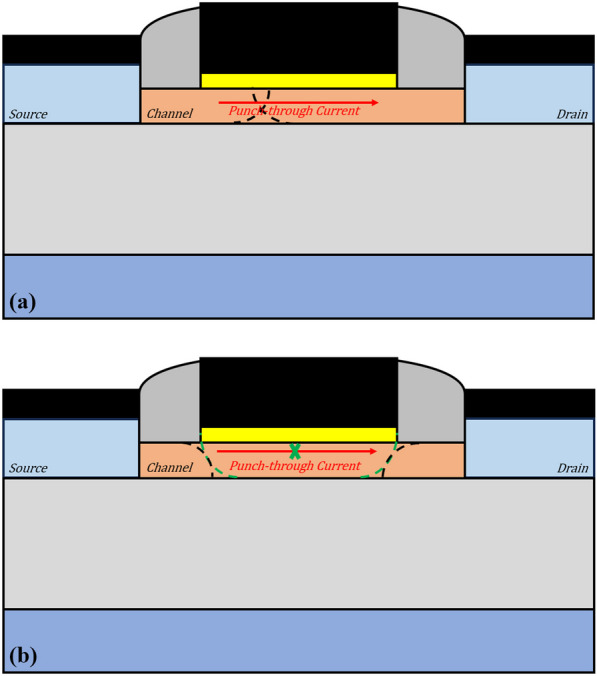
**a** When the PTnMOS is operated in accumulation mode, and **b** When the PTnMOS is operated in weak inversion mode

Figure 2 shows a schematic diagram of the operation of the PTnMOS. According to Fu-Chieh Hsu et al. [21], when the device is operated in accumulation mode (gate bias (*V*_G_) is below the flat-band voltage (*V*_FB_)), punch-through occurs below the surface (i.e., the lowest barrier path seen by the electrons between the source and the drain is below the surface). The magnitude of the current is determined by the minimum potential value along this path, resulting in a punch-through current as shown in Fig. 2a. The black dotted lines in Fig. 2a represent the depleted region of the source/channel and the drain/channel, respectively. As a result, PTnMOS operate with a negative gate bias, providing electrical characteristics similar to those of pMOS. When the device is operated in weak inversion mode (gate bias is above the flat-band voltage but below the threshold voltage (*V*_th_)), depleted region is formed between gate oxide and channel. As the gate bias increases, the depletion region between the gate and the channel expands. This results in the separation of the depletion region between the source/channel and the drain/channel, preventing the formation of a punch-through current and leading to a closed state, as shown in Fig. 2b. The green dotted line in Fig. 2b represents the depletion region between the gate oxide and the channel.

**Fig. 3:**
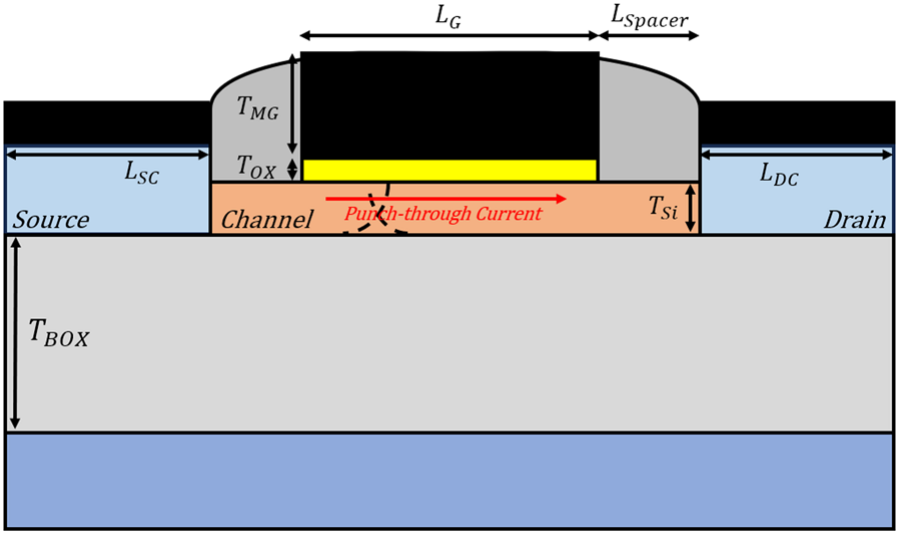
Schematic of PTnMOS structure

**Fig. 4:**
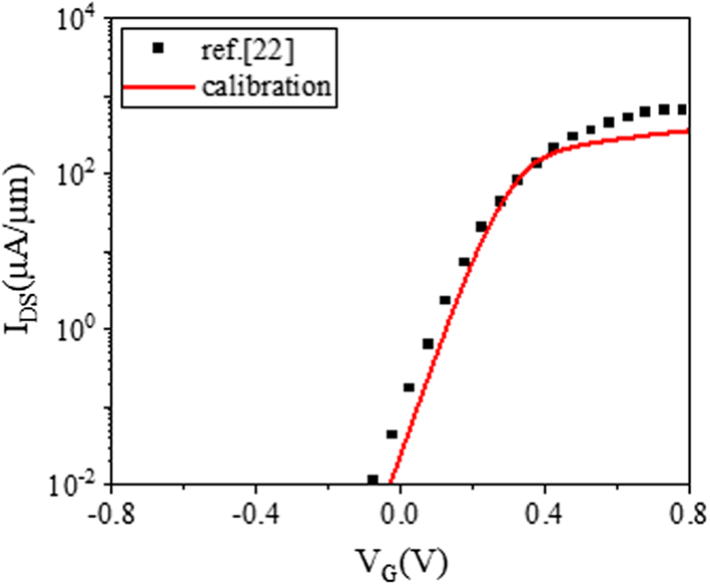
Calibration plot of simulation parameters for the PTnMOS with zero back gate voltage

Table 1 shows the device parameters of our proposed PTnMOS, and device structure shown in Fig. 3. In order to match our proposed PTnMOS with the results of real fabrication measurements, we calibrated the 22 nm FD SOI presented by Carter et al. [22] at IEDM 2016 using Silvaco Athena [23] and Atlas [24]. Considering various carrier generation-recombination models, including SRH, CONSRH, and AUGER. Additionally, we considered the impact ionization model IMPACT SELB. The thinness of the gate insulation layer may cause potential gate leakage current issues. To consider this, models such as BBT.KL, FNORD, and FNHOLES were used. We also considered carrier mobility models such as CONMOB, FLDMOB, and CVT, as well as Fermi and BGN carrier statistics models. Figure 4 shows the results of calibrating the PTnMOS using the aforementioned model. It is evident that the model parameters we have employed closely resemble the actual situation.

**Table 1:** Device parameters used for PTnMOS

Device parameters	
Burrier oxide thickness (*T*_*BOX*_)	100 nm
Semiconductor thickness (*T*_*Si*_)	10 nm
Gate length (*L*_G_)	22 nm
Gate oxide thickness (*T*_OX_)	3 nm
Metal gate thickness (*T*_MG_)	20 nm
Source contact length (*L*_SC_)	11 nm
Drain contact length (*L*_DC_)	11 nm
Side wall spacer length (*L*_Spacer_)	11 nm
Channel concentration (*p*-type)	1 × 10^17^ cm^−3^
Source/drain concentration (*n*-type)	2 × 10^17^ cm^−3^

## Result and discussion

### Effect of changing source/drain concentration on PTnMOS.

**Fig. 5:**
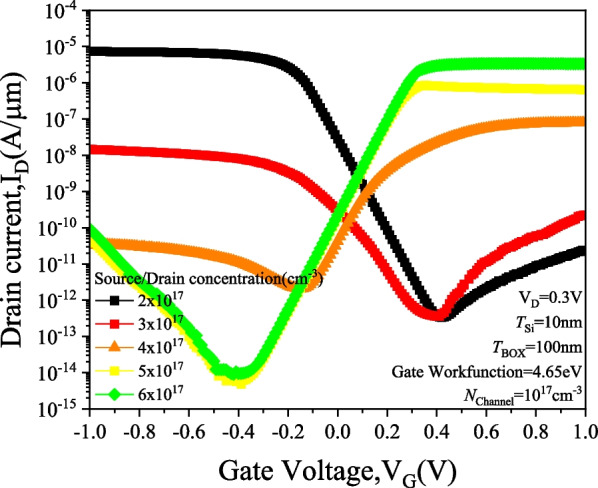
*I*_D_–*V*_G_ characteristics of PTnMOS changing source/drain concentrations

**Fig. 6:**
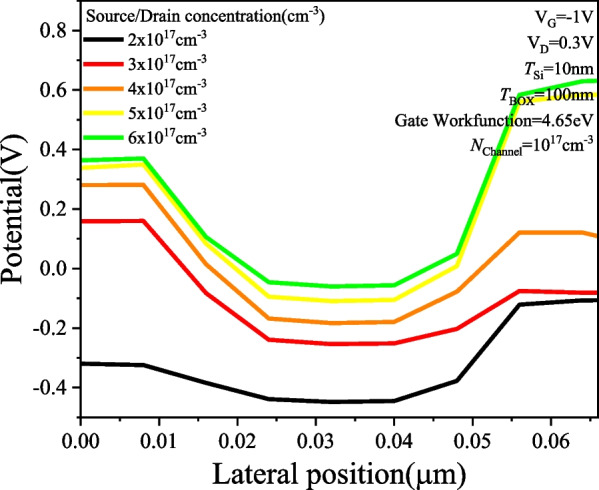
Potential characteristics of PTnMOS with varying source/drain concentrations

**Fig. 7:**
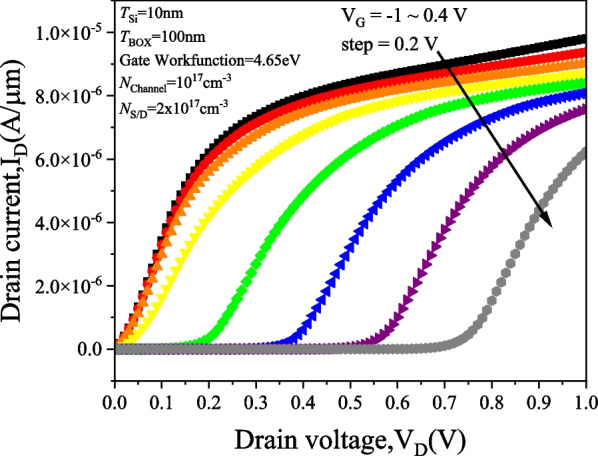
Output characteristics of PTnMOS

Figure 5 shows the *I*_D_–*V*_G_ characteristics of PTnMOS with different source/drain concentrations at a fixed channel concentration. The gate workfunction is set at 4.65 eV (i.e. *V*_FB_ = 0 V) while the source/drain concentration is adjusted to achieve punch-through. The results indicate that PTnMOS can be fabricated when the source/drain concentration is approximately 2 to 3 times higher than the channel concentration, and its electrical properties are similar to those of pMOS. From the source/drain concentration of 2 × 10^17^ cm^−3^, it can be found that when *V*_G_ < *V*_FB_ (negative gate bias), the depletion region between source/channel and drain/channel touch each other, resulting in a punch-through current that reaches the on-state. When *V*_FB_ < *V*_G_ < *V*_th_ (gate bias is positive), the depletion region of gate oxide/channel grows with increasing gate bias, pushing the depletion region of source/channel away from the depletion region of drain/channel to block the punch-through current and reach the off-state. Figure 6 shows the potential of PTnMOS to change the source/drain concentration at V_G_ =  − 1 V. It can be observed that the punch-through current is higher for the lower barrier paths when the PTnMOS is operated in the accumulation mode. This is consistent with what we mentioned in Section III, that the punch-through current is determined by the value of the minimum potential along the path. Figure 7 illustrates the output characteristics of PTnMOS. It can be observed that the punch-through current decreases when *V*_G_ increases.

### PTnMOS operating modes

**Fig. 8:**
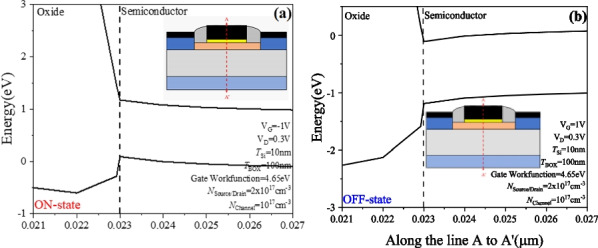
**a** The PTnMOS operates in the accumulation mode when *V*_G_ =  − 1 V, and **b** it operates in the weak inversion mode when *V*_G_ = 1 V

**Fig. 9:**
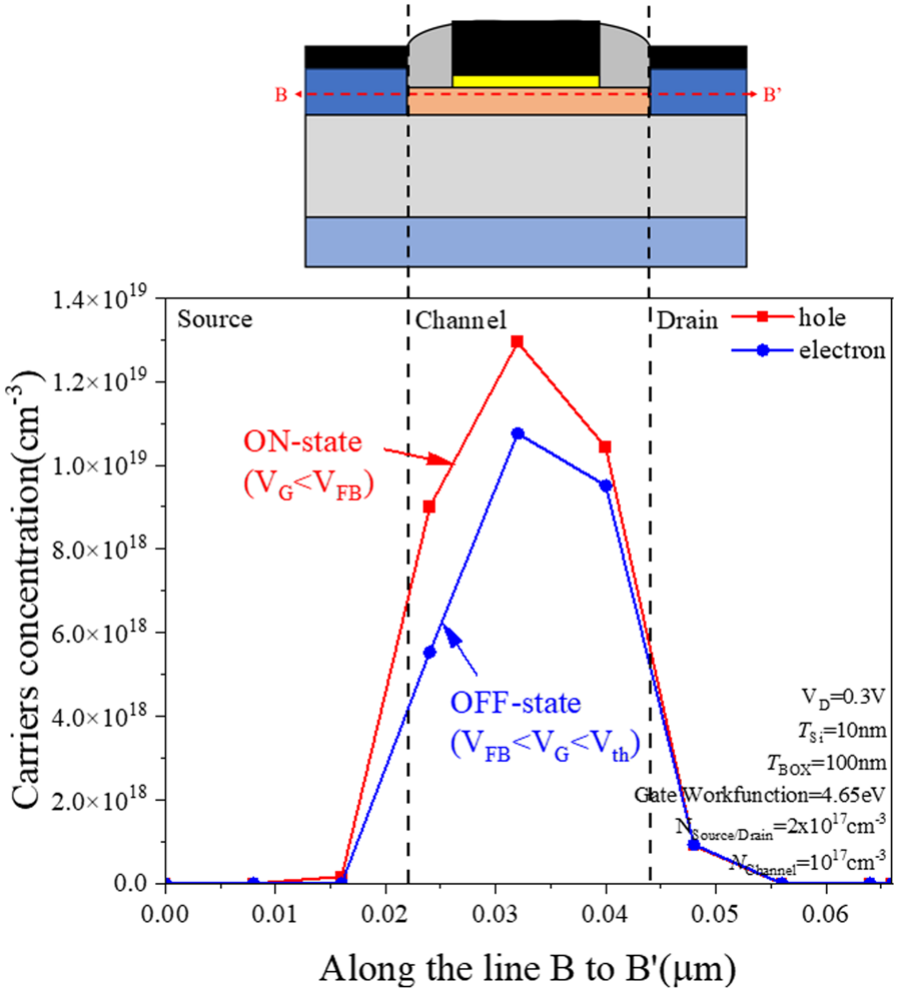
Carrier concentration under the gate oxide when the PTnMOS is operated in the on and off states

Figure 8 shows the energy band diagram at different gate voltages. To observe the operating mode of the PTnMOS, we fixed the channel concentration at 10^17^ cm^−3^ and the source/drain concentration at 2 × 10^17^ cm^−3^. We observe the changes of the energy band diagrams along A to A′. Figure 8a shows that when *V*_G_ =  − 1 V, the energy band of the oxide and semiconductor interface is upward curved and the same as that of the nMOS operation in the accumulating mode. This indicates that the PTnMOS operation is turned on in the accumulating mode. Then we observe the energy band diagram at *V*_G_ = 1 V as shown in Fig. 8b, the energy band of the oxide and semiconductor interface is downward curved, which is the same as that of nMOS operation in weak inversion mode, indicates that the PTnMOS operation in weak inversion mode is turned off. Figure 9 shows the carrier concentration under the gate oxide of PTnMOS at different gate bias voltages. The change in carrier concentration along B to B' can be observed. When the PTnMOS is operating in the ON-state (*V*_G_ =  − 1 V), a large number of holes are attracted under the gate oxide. Conversely, in the OFF-state (*V*_G_ = 1 V), a large number of electrons are attracted under the gate oxide of the PTnMOS. This phenomenon is identical to that of nMOS. Because the PTnMOS operation is ON-state during the accumulation mode, we are able to form a single-carrier CMOS with only electronic transmission with two nMOS device.

### Optimized electrical characteristics of PTnMOS

**Fig. 10:**
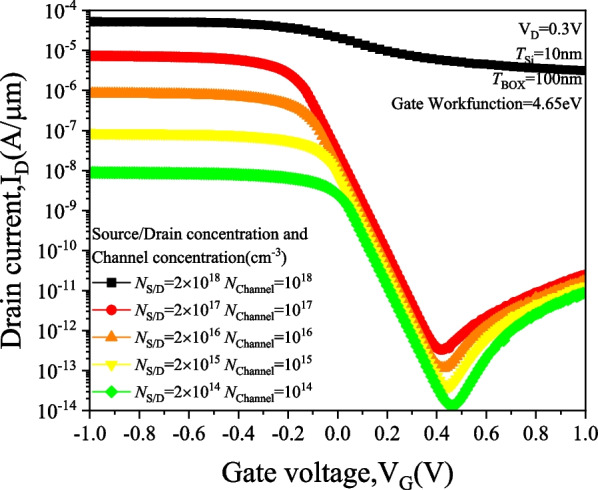
*I*_D_–*V*_G_ characteristic diagram for increasing the source/drain and channel concentration at the same time under the punch-through effect

**Fig. 11:**
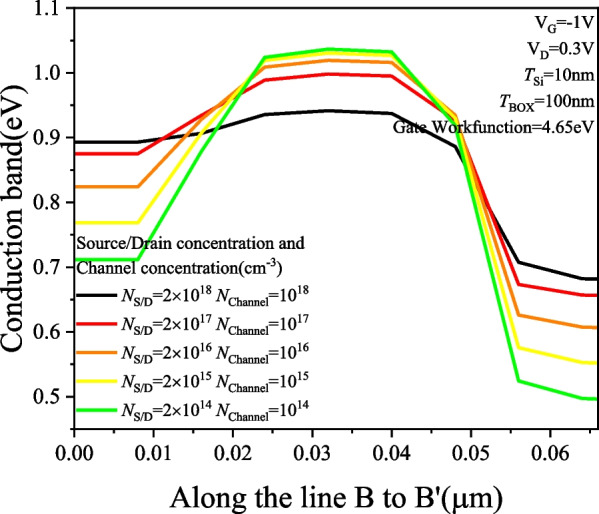
Conduction band diagrams with simultaneous increase in source/drain and channel concentration while maintaining the punch-through effect

Figure 10 illustrates the *I*_D_–*V*_G_ characteristics of the PTnMOS in detail, showing that the punch-through current increases with the carrier concentration. It can be observed that the punch-through current of the PTnMOS is directly proportional to the concentration, and the punch-thourgh current increases by one order for each order of the carrier concentration; however, the ability to turn off the punch-through effect is reduced when the channel concentration reaches 10^18^ cm^−3^ and the source/drain concentration reaches 2 × 10^18^ cm^−3^, which deteriorates the electrical properties of the PTnMOS. From the conduction band diagram in Fig. 11, it can also be seen that as the concentration increases at *V*_G_ =  − 1 V, the potential barrier between the source and the channel decreases, making it easier for electrons to pass through the barrier. Additionally, higher concentrations allow more electrons to cross the barrier, resulting in an increase in punch-through current.

**Fig. 12:**
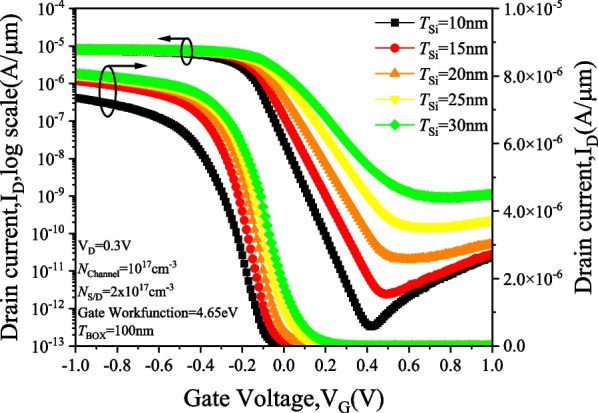
*I*_D_–*V*_G_ characteristics of PTnMOS with varying semiconductor layer thickness

**Fig. 13:**
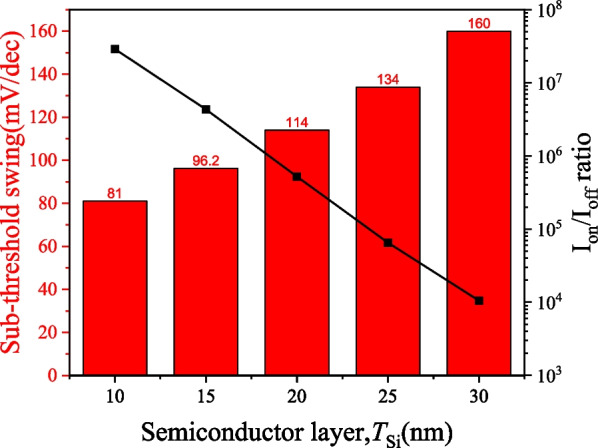
Sub-threshold swing and *I*_on_/*I*_off_ ratio characteristics of PTnMOS with varying semiconductor layer thickness

Figure 12 shows the effect of varying the semiconductor layer thickness on the *I*_D_–*V*_G_ characteristics of the PTnMOS. The sub-threshold swing performance improves as the semiconductor layer thickness decreases. At a semiconductor layer thickness of 10 nm, the sub-threshold swing reaches 81 mV/dec with an *I*_on_/*I*_off_ ratio of 2.89 × 10^7^, as shown in Fig. 13. Reducing the thickness of the semiconductor layer enhances the coverage of the depletion region between the gate oxide layer and the semiconductor layer. The depletion region between the source/channel and the drain/channel can be easily pushed away, preventing the punch-through current from continuing to flow, thus enabling the PTnMOS to achieve better sub-threshold swing and *I*_on_/*I*_off_ ratios. When the semiconductor layer is too thick, it becomes difficult to effectively push away the depletion region between the source/channel and the drain/channel. This leads to difficulty in interrupting the punch-through current, requiring a larger gate bias to increase the depletion region between the gate oxide layer and the semiconductor layer, which in turn interrupts the punch-through current. Consequently, the sub-threshold swing becomes worse and the *I*_on_/*I*_off_ ratio of PTnMOS decreases when body thickness becomes thicker.

### Impact of drain induced barrier lowering-like (DIBL-like) on PTnMOS

**Fig. 14:**
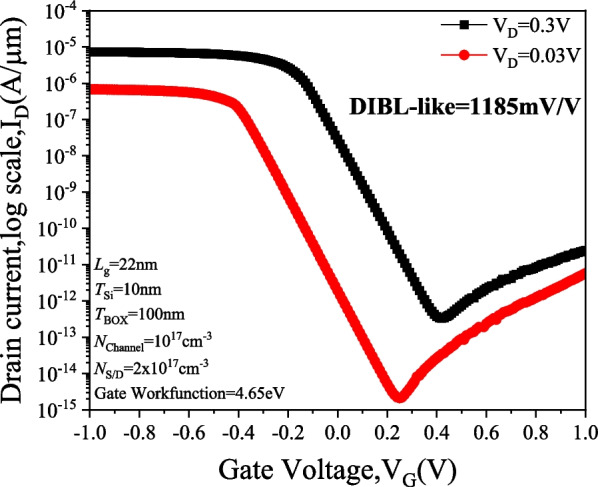
*I*_D_–*V*_G_ characteristics of PTnMOS at *V*_D_ = 0.3 V (saturation region) and *V*_D_ = 0.03 V (linear region)

**Fig. 15:**
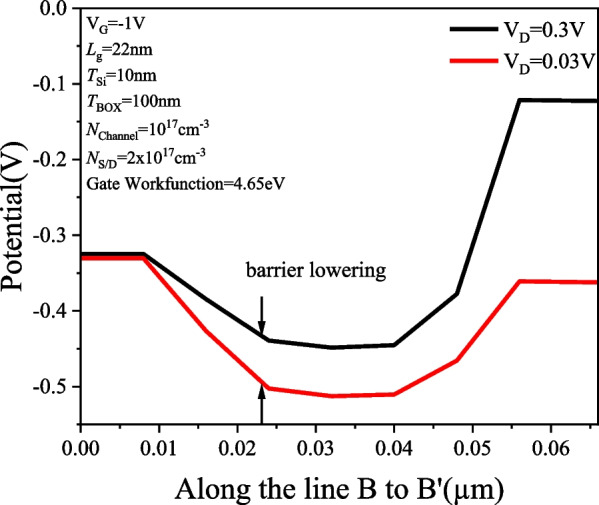
Potential diagram of PTnMOS at *V*_D_ = 0.3 V (saturation region) and *V*_D_ = 0.03 V (linear region)

Figure 14 shows the *I*_D_–*V*_G_ characteristics of the PTnMOS at *V*_D_ = 0.3 V (saturation region) and *V*_D_ = 0.03 V (linear region). It can be seen that the PTnMOS utilizes the punch-through effect to enhance the electron injection by lowering the potential barrier between the source and the channel in the accumulation mode. In addition, as the punch-through effect increases (i.e., the drain bias increases), the more the potential barrier decreases and the punch-through current increases. This behaviour is similar to that of DIBL, but PTnMOS operates based on punch-through current rather than the channel carrier drift current of nMOS. Therefore, we refer to this as DIBL-like. This behavior results in a very large DIBL-like value, calculated to be 1185 mV/V. This value indicates that the output current of the PTnMOS is very sensitive to the voltage between the source and drain, and it also means that the charge time of a PTnMOS with pMOS characteristics is very short when charging, which is advantageous for controlling the charge time of the logic circuit. Figure 15 shows the relationship between *V*_D_ = 0.3 V and *V*_D_ = 0.03 V potential and lateral position. It can clearly see that the potential barriers between the source and the channel are significantly reduced. This therefore emphasizes the importance of the punch-through effect in the operation of punch-through nMOSFET and how it affects the relationship between gate voltage and drain current.

### PTnMOS and nMOS form a single-carrier CMOS

**Fig. 16:**
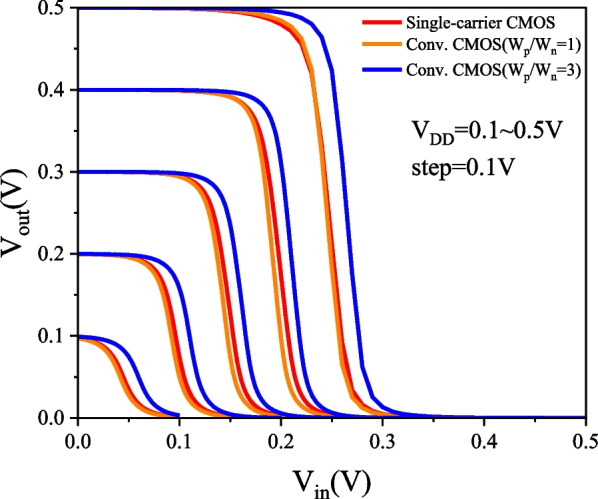
Voltage transfer curves for single-carrier CMOS and conventional CMOS (W_p_/W_n_ = 1) and conventional CMOS (W_p_/W_n_ = 3) circuits at different supply voltages

**Fig. 17:**
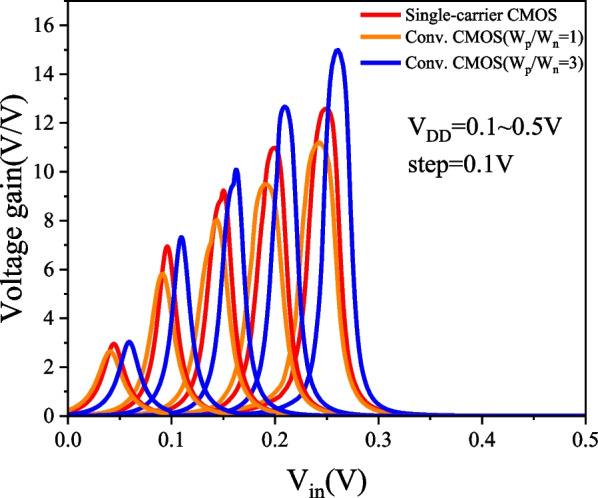
Voltage gain curves for single-carrier CMOS and conventional CMOS (W_p_/W_n_ = 1) and conventional CMOS (W_p_/W_n_ = 3) circuits at different supply voltages

**Fig. 18:**
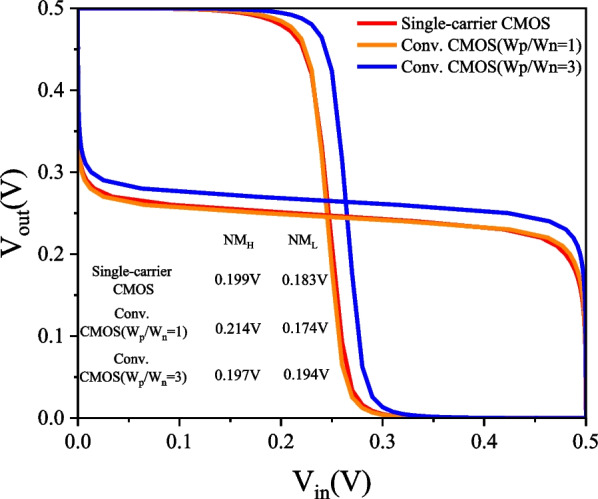
Butterfly curve of single-carrier CMOS and conventional CMOS (W_p_/W_n_ = 1) and conventional CMOS (W_p_/W_n_ = 3) circuits

**Fig. 19:**
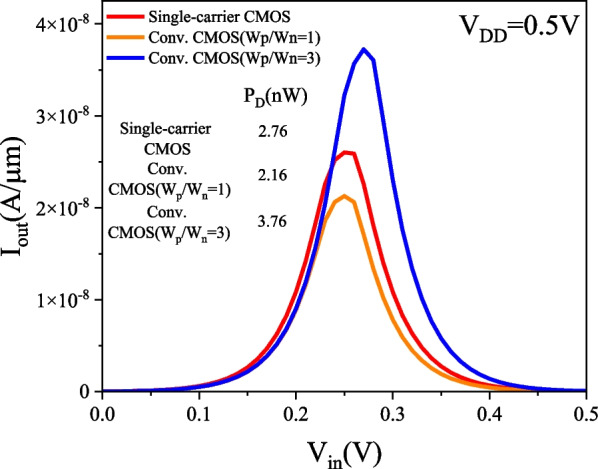
*I*_out_−*V*_in_ transfer characteristics of single-carrier CMOS and conventional CMOS (W_p_/W_n_ = 1) and conventional CMOS (W_p_/W_n_ = 3) circuits

**Fig. 20:**
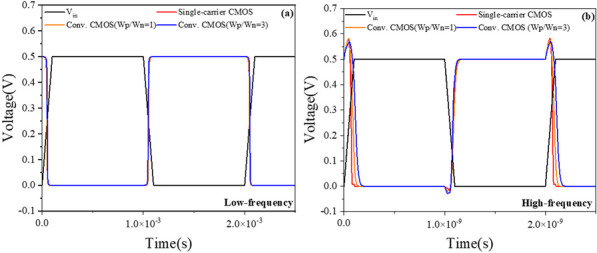
Propagation times of single-carrier CMOS and conventional CMOS (W_p_/W_n_ = 1) and conventional CMOS (W_p_/W_n_ = 3) circuits at low frequencies (**a**) and high frequencies (**b**)

**Fig. 21:**
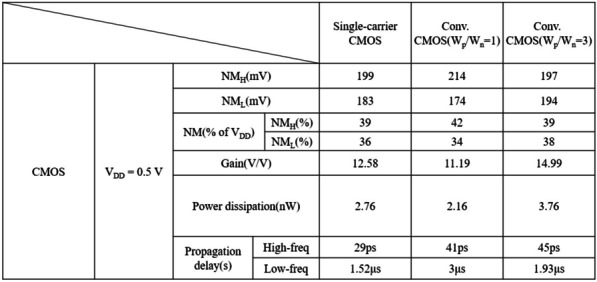
Comparison of results in DC performance between single-carrier CMOS and conventional CMOS (W_p_/W_n_ = 1) and conventional CMOS (W_p_/W_n_ = 3)

We used Silvaco MixedMode to analyse the single-carrier CMOS and compare it with conventional CMOS (W_p_/W_n_ = 1) and conventional CMOS (W_p_/W_n_ = 3). The parameters of nMOS and pMOS are the same as those of PTnMOS. The DC performance of the three inverter circuits is measured by inserting a load capacitance (C_L_), which is the sum of the internal capacitances of the PTnMOS and nMOS for single-carrier CMOS, and the nMOS and pMOS for conventional CMOS. Figure 16 shows the voltage transfer curves of three circuits at different supply voltages. Since the sub-threshold characteristics of the PTnMOS can replace the pMOS in the conventional CMOS circuit, when the PTnMOS is the driver and the nMOS is the load, the single-carrier CMOS circuit can maintain good characteristics under different supply voltages (*V*_DD_). Figure 17 shows the voltage gain of three circuits at different supply voltages. The effect of the pMOS width adjustment is to achieve a ratio of pMOS to nMOS drive current of 1, which results in a slight increase in the voltage gain of conventional CMOS (W_p_/W_n_ = 3) compared to that of single-carrier CMOS. The voltage gains at V_DD_ = 0.5 V are 12.58 for single-carrier CMOS, 11.19 for conventional CMOS (W_p_/W_n_ = 1), and 14.99 for conventional CMOS (W_p_/W_n_ = 3), respectively. Figure 18 shows the butterfly curve of the three circuits at V_DD_ = 0.5 V. The following mathematical equations can be used to calculate high noise margin (*NM*_H_) and low noise margin (*NM*_L_) [25],

1 $$\begin{array}{*{20}c} {{\text{NM}}_{H} = V_{{{\text{OH}}}} - V_{{{\text{IH}}}} } \\ \end{array}$$

2 $$\begin{array}{*{20}c} {{\text{NM}}_{L} = V_{{{\text{IL}}}} - V_{{{\text{OL}}}} } \\ \end{array}$$

the input voltages *V*_IL_ and *V*_IH_ are the voltages with voltage gain *dV*_out_/*dV*_in_ =  − 1 in Fig. 18. *V*_OL_ and *V*_OH_ can also be calculated from the transfer characteristics by considering the minimum and maximum voltage values. Calculated from Eqs. (1) and (2), *NM*_H_ and *NM*_L_ are 0.199 V and 0.183 V for single carrier CMOS. For conventional CMOS (W_p_/W_n_ = 1) they are 0.214 V and 0.174 V. For conventional CMOS (W_p_/W_n_ = 3) they are 0.197 V and 0.194 V. The percentages of V_DD_ for single-carrier CMOS are 39% and 36%, while for conventional CMOS (W_p_/W_n_ = 1) they are 42% and 34%, and for conventional CMOS (W_p_/W_n_ = 3) they are 39% and 38%. It can be found that single-carrier CMOS exhibits symmetric noise margin compared to conventional CMOS (W_p_/W_n_ = 1). Figure 19 shows the *I*_out_–*V*_in_ characteristics of the three circuits at V_DD_ = 0.5 V. The static power dissipation (*P*_D_) was calculated to be 2.76 nW for single-carrier CMOS, 2.16 nW for conventional CMOS (W_p_/W_n_ = 1), and 3.76 nW for conventional CMOS (W_p_/W_n_ = 1). It can be observed that the static power dissipation of single-carrier CMOS is lower than that of conventional CMOS (W_p_/W_n_ = 3). Figure 20 shows the propagation time of the three circuits at low and high frequencies. In Fig. 20a the delay time at low frequency is calculated to be 1.52us for single carrier CMOS, 3us for conventional CMOS (W_p_/W_n_ = 1) and 1.93us for conventional CMOS (W_p_/W_n_ = 3). The results show that the delay time of single-carrier CMOS is 49% faster than conventional CMOS (W_p_/W_n_ = 1) and is 21% faster than conventional CMOS (W_p_/W_n_ = 3). And the high frequency delay time calculated in Fig. 20b is 29 ps for single carrier CMOS, 41 ps for conventional CMOS (W_p_/W_n_ = 1), and 45 ps for conventional CMOS (W_p_/W_n_ = 3). The results indicate that the delay time of single-carrier CMOS is 29% faster than that of conventional CMOS (W_p_/W_n_ = 1) and 35% faster than conventional CMOS (W_p_/W_n_ = 3). Figure 21 shows the DC performance comparison of three circuits. The results indicate that single-carrier CMOS exhibits superior DC performance compared to conventional CMOS (W_p_/W_n_ = 1). This is due to the fact that the ratio of drive current between PTnMOS and nMOS is about 0.84 times, which is higher than the ratio of drive current between pMOS and nMOS, and the optimised PTnMOS has better electrical properties than the conventional pMOS.

## Conclusion

We have proposed the use of PTnMOS to replace pMOS in conventional CMOS circuits. The operation mechanism and modes of PTnMOS have been explored to optimize their electrical performance. Additionally, the effect of DIBL-like on PTnMOS has been discussed. We realized single-carrier CMOS circuits without sizing and with only electron transmission. The single-carrier CMOS exhibits symmetrical noise margin and 29% faster delay time than conventional CMOS (W_p_/W_n_ = 1). If single-carrier CMOS circuits are transformed into III–V group materials, II–VI group materials, or even two-dimensional materials, sizing becomes unnecessary, thereby resolving the issue of occupied area. The electrical performance of single-carrier CMOS circuits is greatly enhanced by the advantage of ultra-high electron mobility of these materials, making them suitable for modern low-voltage, low-power and high-speed switching CMOS circuits.

## Data Availability

The data and analyses generated in this study have been agreed to by all authors and are available from the corresponding authors upon reasonable request.
